# Modified Q-type purse-string suture duodenal stump embedding method for laparoscopic gastrectomy for gastric cancer

**DOI:** 10.1186/s12893-024-02423-1

**Published:** 2024-04-24

**Authors:** Longhe Sun, Wei Wang, Jiajie Zhou, Lili Ji, Shuai Zhao, Yayan Fu, Ruiqi Li, Jie Wang, Chunhua Qian, Qiannan Sun, Daorong Wang

**Affiliations:** 1 Clinical Medical College, Yangzhou University, Jiangsu, 225001 China; 2 The Forth People’s Hospital of Taizhou, Taizhou, 225300 China; 3 Northern Jiangsu People’s Hospital, Yangzhou, 225001 China; 4 Northern Jiangsu People’s Hospital, Clinical Teaching Hospital of Medical School, Nanjing University, Yangzhou, 225001 China; 5 Northern Jiangsu People’s Hospital, Clinical Medical College of Yangzhou University, Jiangsu, 225001 China; 6 Yangzhou University, Yangzhou Institute of General Surgery, Jiangsu, 225001 China; 7 Yangzhou Key Laboratory of Basic and Clinical Transformation of Digestive and Metabolic Disease, Jiangsu, 225001 China

**Keywords:** Complication, Duodenal stump leakage, Gastric Cancer, Laparoscopic gastrectomy, The Q-type purse-string suture duodenal stump embedding method

## Abstract

**Objective:**

This study introduced the modified Q-type purse-string suture duodenal stump embedding method, a convenient way to strengthen the duodenum, and compared it to the conventional one to assess its efficacy and safety.

**Methods:**

This retrospective analysis examined 612 patients who received laparoscopic gastrectomy for gastric Cancer at a single center. The patients were divided into Not Reinforced Group (*n* = 205) and Reinforced Group (*n* = 407) according to the surgical approach to the duodenal stump. The reinforced group was further divided into a modified Q-type purse-string suture embedding method group (QM, *n* = 232) and a conventional suture duodenal stump embedding method group (CM, *n* = 175) according to the methods of duodenal stump enhancement. Clinicopathological characteristics, operative variables, and short-term complications were documented and analyzed.

**Results:**

The incidence of duodenal stump leakage(DSL) in the Not Reinforced Group was higher compared to the Reinforced Group, although the difference was not statistically significant [2.4% (5/205) vs 0.7% (3/407), *p* = 0.339]. Additionally, the Not Reinforced Group exhibited a higher rate of Reoperation due to DSL compared to the Reinforced Group [2 (1.0%) vs. 0, *p* = 0.046], with one patient in the Not Reinforced Group experiencing mortality due to DSL [1 (0.5%) vs 0, *p* = 0.158]. Subgroup analysis within the Reinforced Group revealed that the modified Q-type purse-string suture embedding group (QM) subgroup demonstrated statistically significant advantages over the conventional suture embedding group (CM) subgroup. QM exhibited shorter purse-string closure times (4.11 ± 1.840 vs. 6.05 ± 1.577, *p* = 0.001), higher purse-string closure success rates (93.1% vs. 77.7%, *p* = 0.001), and greater satisfaction with purse-string closure [224 (96.6%) vs 157 (89.7%), *p* = 0.005]. No occurrences of duodenal stump leakage were observed in the QM subgroup, while the CM subgroup experienced two cases [2 (1.1%)], though the difference was not statistically significant. Both groups did not exhibit statistically significant differences in secondary surgery or mortality related to duodenal stump leakage.

**Conclusion:**

Duodenal Stump Leakage (DSL) is a severe but low-incidence complication. There is no statistically significant relationship between the reinforcement of the duodenal stump and the incidence of DSL. However, laparoscopic reinforcement of the duodenal stump can reduce the severity of fistulas and the probability of Reoperation. The laparoscopic Q-type purse-string suture duodenal stump embedding method is a simple and effective technique that can, to some extent, shorten the operation time and enhance satisfaction with purse-string closure. There is a trend towards reducing the incidence of DSL, thereby improving patient prognosis to a certain extent.

**Supplementary Information:**

The online version contains supplementary material available at 10.1186/s12893-024-02423-1.

## Introduction

Currently, the primary therapeutic approach for gastric Cancer remains comprehensive treatment with surgery as the cornerstone [1, 2]. Laparoscopic gastrectomy combined with lymph node dissection is the standard treatment for early-stage gastric cancer [3–5], and is also a selectable surgical option for advanced gastric cancer [6, 7]. Due to its minimal invasiveness and rapid recovery, laparoscopic surgery is increasingly being widely applied clinically. Particularly in East Asia, it has become the predominant treatment modality for gastric cancer [8].

In the spectrum of complications observed in gastric cancer surgery patients, Duodenal Stump Leakage (DSL), or duodenal stump leakage, though a rare occurrence, stands out as one of the most severe complications with a reported failure rate of conservative treatment at 30% and a high mortality rate of up to 28% [9]. Furthermore, there is a lack of a standardized treatment approach suitable for all cases [10]. Studies indicate that laparoscopic gastric cancer surgery increases the risk of duodenal stump leakage [10], prompting some investigations to explore various methods of duodenal stump reinforcement to mitigate this risk [11–15]. However, conflicting evidence exists, with some studies suggesting that routine reinforcement of the duodenal stump is unnecessary for curative gastric cancer surgery [15, 16]. The controversy surrounding whether to reinforce the duodenal stump and, if so, how to achieve optimal reinforcement with minimal time and simplified steps warrants further exploration.

In this context, our center has undertaken a modification of the conventional duodenal stump reinforcement method, termed the modified Q-type purse-string suture duodenal stump embedding method. This study retrospectively analyzes clinical and pathological data from patients who underwent laparoscopic radical gastrectomy at our center, aiming to explore the advantages of the Q-type suture technique in clinical practice.

## Patients and methods

### Patients

This study prospectively enrolled consecutive patients with gastric Cancer, collecting clinical and pathological data. The research focused on patients who underwent laparoscopic radical total gastrectomy (TLTG) with Roux-en-Y anastomosis or radical distal gastrectomy (TLDG) with Billroth II + Braun anastomosis for gastrointestinal reconstruction between January 2019 and August 2023 at the Gastrointestinal Center of Northern Jiangsu People's Hospital and The Forth People's Hospital. A retrospective analysis was conducted on clinical and pathological data, surgical details, and short-term postoperative complications. Ethical Approval for this study was obtained from the local institutional ethics committee, and all patient information in this study was handled anonymously.

### Inclusion and exclusion criteria

Inclusion Criteria: (1) Laparoscopic radical gastrectomy with Roux-en-Y anastomosis, Billroth II + Braun anastomosis for gastrointestinal reconstruction. (2) Patients with histopathologically confirmed primary gastric Cancer. (3) Absence of tumor metastasis at the time of surgical resection. (4) Age range between 18 and 75 years.

Exclusion Criteria: (1) Difficult closure of duodenal stump. (2) Tumor invasion into the pancreatic-duodenal area. (3) Patients undergoing simultaneous resection of multiple lesions. (4) Patients receiving neoadjuvant chemoradiotherapy or targeted therapy before surgery. (5) Incomplete clinical and medical records.

### Grouping

Based on the different surgical approaches for the duodenal stump, the participants were divided into two groups: the Not Reinforced Group and the Reinforced Group. Within the Reinforced Group, subgroups were further defined based on the method of duodenal stump reinforcement. Specifically, the Reinforced Group was subdivided into two subgroups: the modified Q-type purse-string suture duodenal stump embedding method group (QM) and the conventional suture duodenal stump embedding method group (CM).

### Surgical procedure

The patient was positioned in the reverse Trendelenburg position with legs apart. The primary surgeon stood on the left" side of the patient, while an assistant stood on the right side, and the camera holder was positioned between the patient's legs. A 1.0 cm transverse incision was made below the umbilicus, and a 10 cm trocar was inserted as an observation port to establish pneumoperitoneum (pressure: 11–13 mmHg). A 1.2 cm incision at the intersection of the left" rib margin and the midline of the left" clavicle served as the main operating port. Additionally, 0.5 cm incisions were made: one at the intersection of the right rib margin and the midline of the right clavicle, and the other 8 cm above and to the left" of the umbilicus, as well as 8 cm above and to the right of the umbilicus, serving as auxiliary operating ports. Standard laparoscopic radical gastrectomy with D1 + or D2 lymph node dissection was performed. For cancers in the upper and middle parts of the stomach, radical total gastrectomy with Roux-en-Y anastomosis was conducted(The esophagojejunostomy was performed laparoscopically using the Overlap technique, while the anastomosis between the jejunal segments was performed side-to-side). For cancers in the distal part of the stomach, radical distal gastrectomy with Billroth II anastomosis and Braun anastomosis were performed(The gastroduodenostomy was performed laparoscopically using a linear cutting closure device for posterior wall gastroduodenal anastomosis, while the Braun anastomosis between the jejunal segments was performed side-to-side). The linear cutting closure device (staple length 6 cm, closure height 3.5 mm) was used to cut and close the duodenum 2–3 cm below the pylorus (Fig. 2A). In the event of instrument malfunction, a 3–0 absorbable suture with needle will be utilized for full-thickness continuous suturing of the duodenal stump (rare occurrence).

#### Not reinforced group surgery procedure

The examination of the duodenal stump was concluded following electrocoagulation to address any bleeding or oozing sites.

#### The conventional suture duodenal stump embedding method (Fig. 1)

**Fig. 1:**
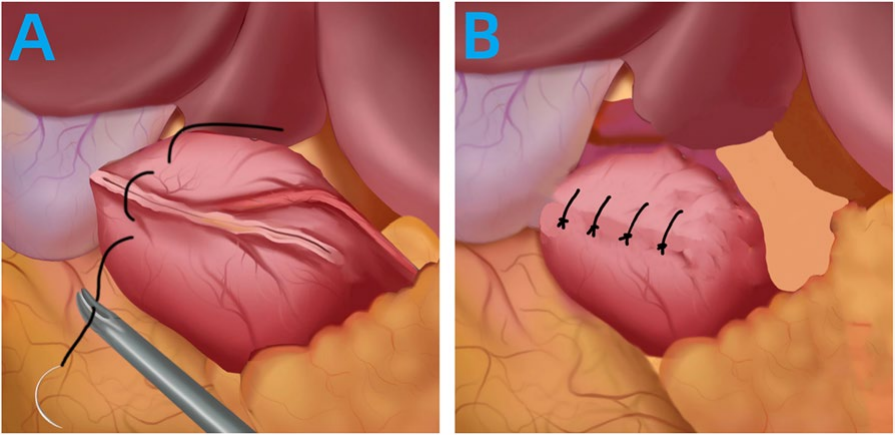
Surgical Procedure for the conventional suture duodenal stump embedding method

Intermittent sutures were placed on both sides of the closure line, involving 4–5 stitches in the muscular layers using 3–0 absorbable suture with needle (Fig. 1A). The sutures were tightened, burying the duodenal stump (Fig. 1B).

#### Laparoscopic modified Q-type purse-string suture duodenal stump embedding method (Fig. 2, see video)

**Fig. 2:**
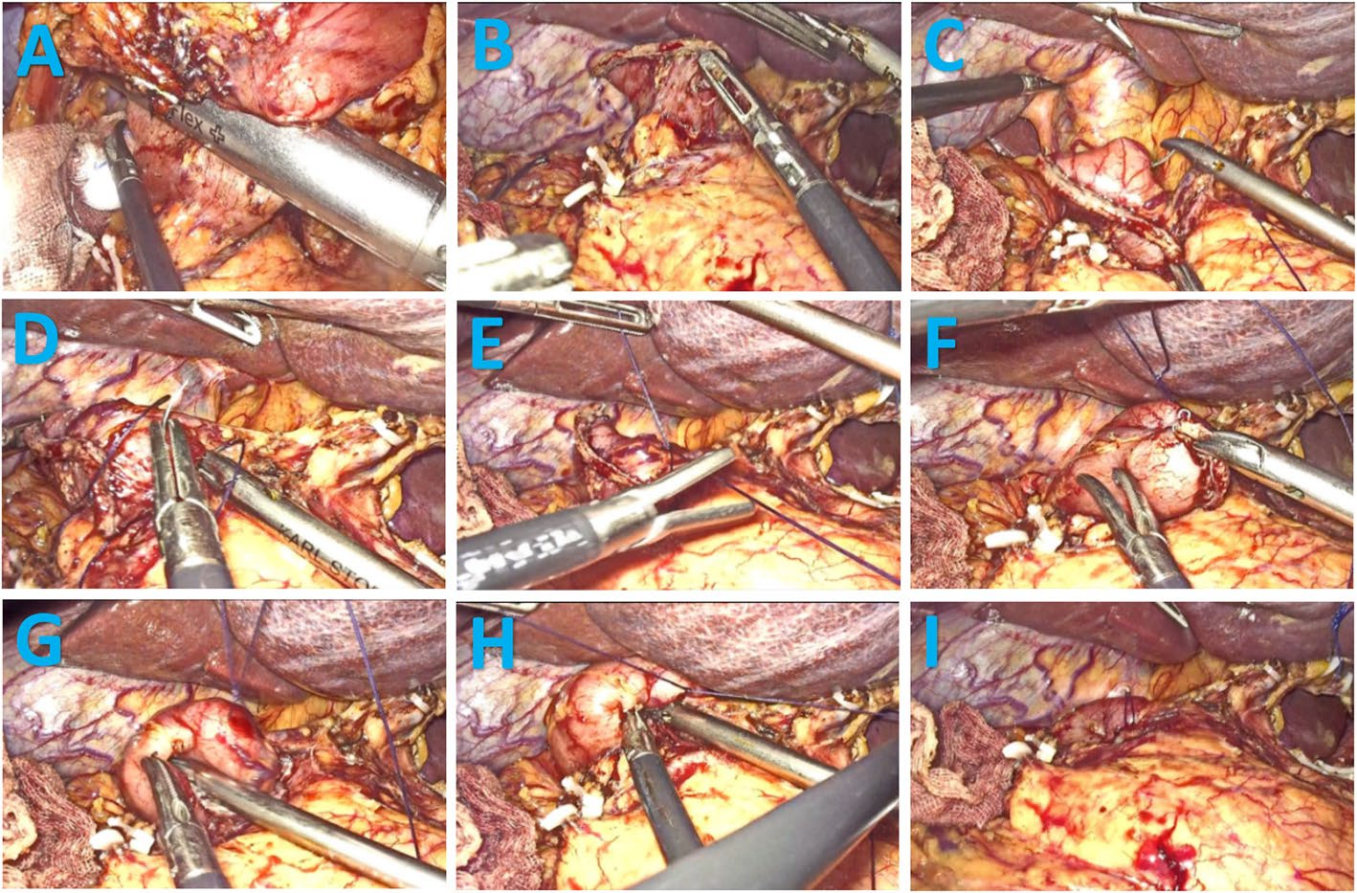
Surgical Procedure for laparoscopic modified Q-type purse-string suture duodenal stump embedding method

Preserving approximately 2–3 cm of the duodenal stump (Fig. 2B), the first stitch was made about 1–1.5 cm from the closure line above the duodenal vascular bundle (Fig. 2C). Sequential sutures were then placed at five locations: the upper, right, lower, and left" sides of the duodenal stump, creating a purse-string formation (Fig. 2D). The surgeon temporarily left" the suture loose after knotting it and handed it over to the assistant to pull up the two threads (Fig. 2E). The surgeon's left" hand used separation forceps to grasp the right-side thread of the purse string as the leverage edge (Fig. 2F). Simultaneously, the surgeon's right hand, using a needle holder, inserted one corner of the duodenal stump into the purse string (Fig. 2G). With coordinated movements, the surgeon inserted the remaining duodenal stump into the purse string, and the assistant tightened the purse string (Fig. 2H). The process continued with knotting and reinforcing the purse string until the burial of the purse was complete (Fig. 2I).

#### Schematic diagram of laparoscopic modified Q-type purse-string suture duodenal stump embedding method (Fig. 3)

**Fig. 3:**
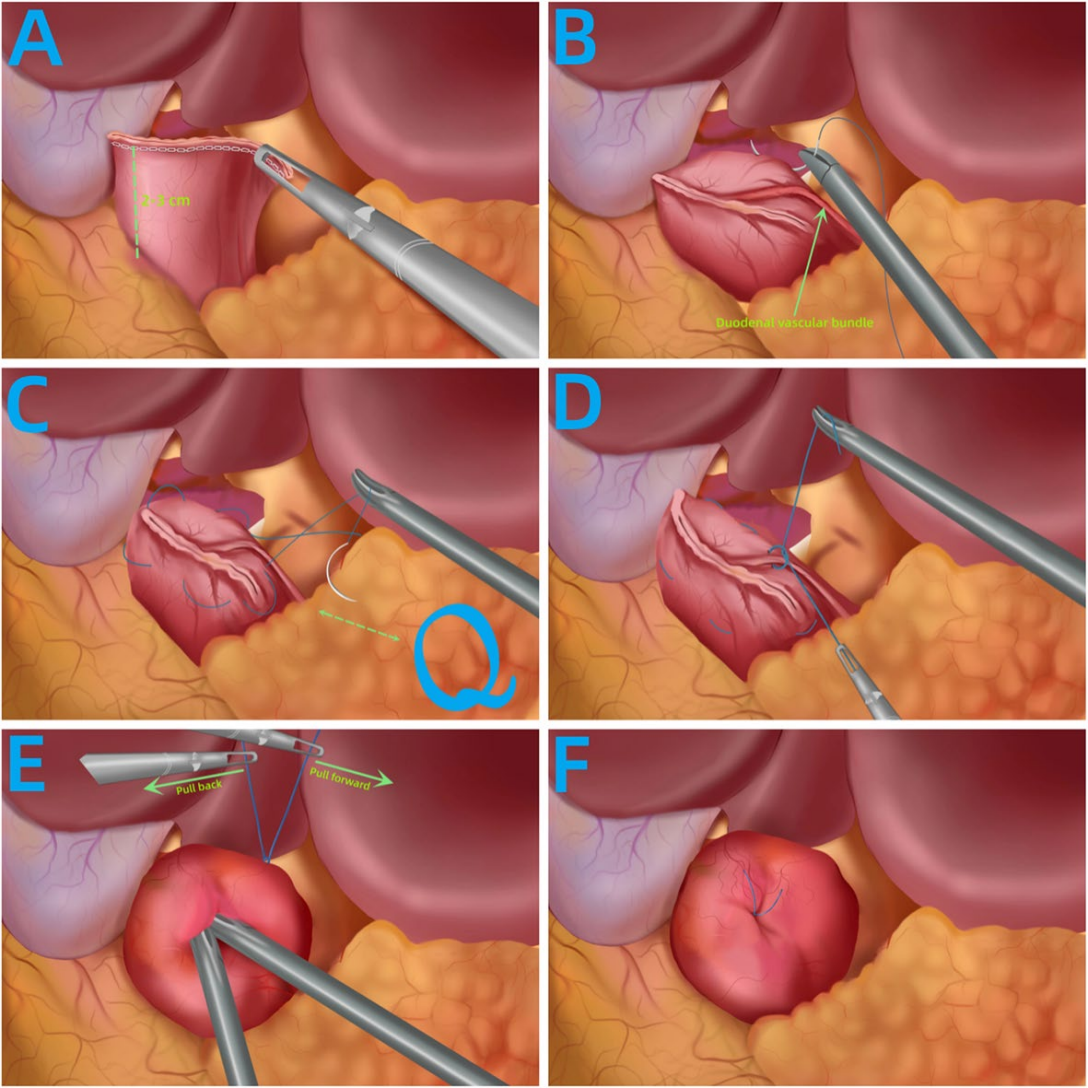
Schematic diagram of the Q-type purse-string suture duodenal stump embedding method

(A) Using a 6 cm linear cutting stapler, cut and close the duodenum 2–3 cm below the pylorus, preserving approximately 3 cm of the duodenal stump (Fig. 3A).
(B) The first stitch was made about 1–1.5 cm above the duodenal vascular bundle from the closure line (Fig. 3B).
(C) Starting 1–1.5 cm above the closure line, a counterclockwise purse-string suture was performed, sequentially suturing five locations on the duodenal stump vascular bundle: left" sides above the duodenal vascular bundle, above, upper, right, below, and below the vascular bundle. The suture forming a circle resembled the letter Q (Fig. 3C).
(D) The surgeon temporarily left" the knotted suture loose and transferred it to the assistant (Fig. 3D).
(E) The assistant lifted the suture, and with coordinated movements, the surgeon inserted the entire duodenal stump into the purse string. The assistant tightened the purse string (Fig. 3E).
(F) Continuing with knotting and reinforcing the purse string, the burial of the purse was completed.

This method was named laparoscopic modified Q-type purse-string suture duodenal stump embedding method due to the circular shape resembling the letter Q in the suture pattern, as well as the modified suture sequence and emphasized details of assistant involvement (Fig. 3F).

#### Innovations

1. Preservation of Duodenal Vascular Bundle:
  During denudation of the duodenum, preservation of duodenal vascular bundles is typically performed to ensure optimal blood supply to the stump (Fig. 3B). However, existing literature on duodenal reinforcement techniques often overlooks how to handle duodenal vascular bundles during suturing. Inevitably, suturing may cause damage to these delicate vessels, resulting in bleeding. This undoubtedly increases surgical time and diminishes procedural smoothness. This study emphasizes the protection of duodenal vascular bundles during needle insertion and withdrawal points to avoid collateral damage, thus enhancing procedural smoothness.
2. Detailed Operational Techniques:
  While various reinforcement methods have been mentioned in previous studies, specific operational details and how the assistant should collaborate have often been overlooked. Some seemingly simple operations carry a certain failure rate. This study emphasizes specific operational details, focusing on improving the success rate of a single burying procedure, avoiding repeated operations, and minimizing collateral damage. The introduction of the satisfaction level of duodenal burial is also quantitatively analyzed to evaluate the advantages and disadvantages of the modified and conventional methods.
3. Assistant Collaboration in Duodenal Burial:
  When burying the duodenal stump, the operative space is constrained, and precise maneuvers are required. This study elaborates on the details of how the assistant collaborates with the surgeon during duodenal stump burial, significantly improving the smoothness of the burial process and reducing the burial time.

### Data acquisition

**Fig. 4:**
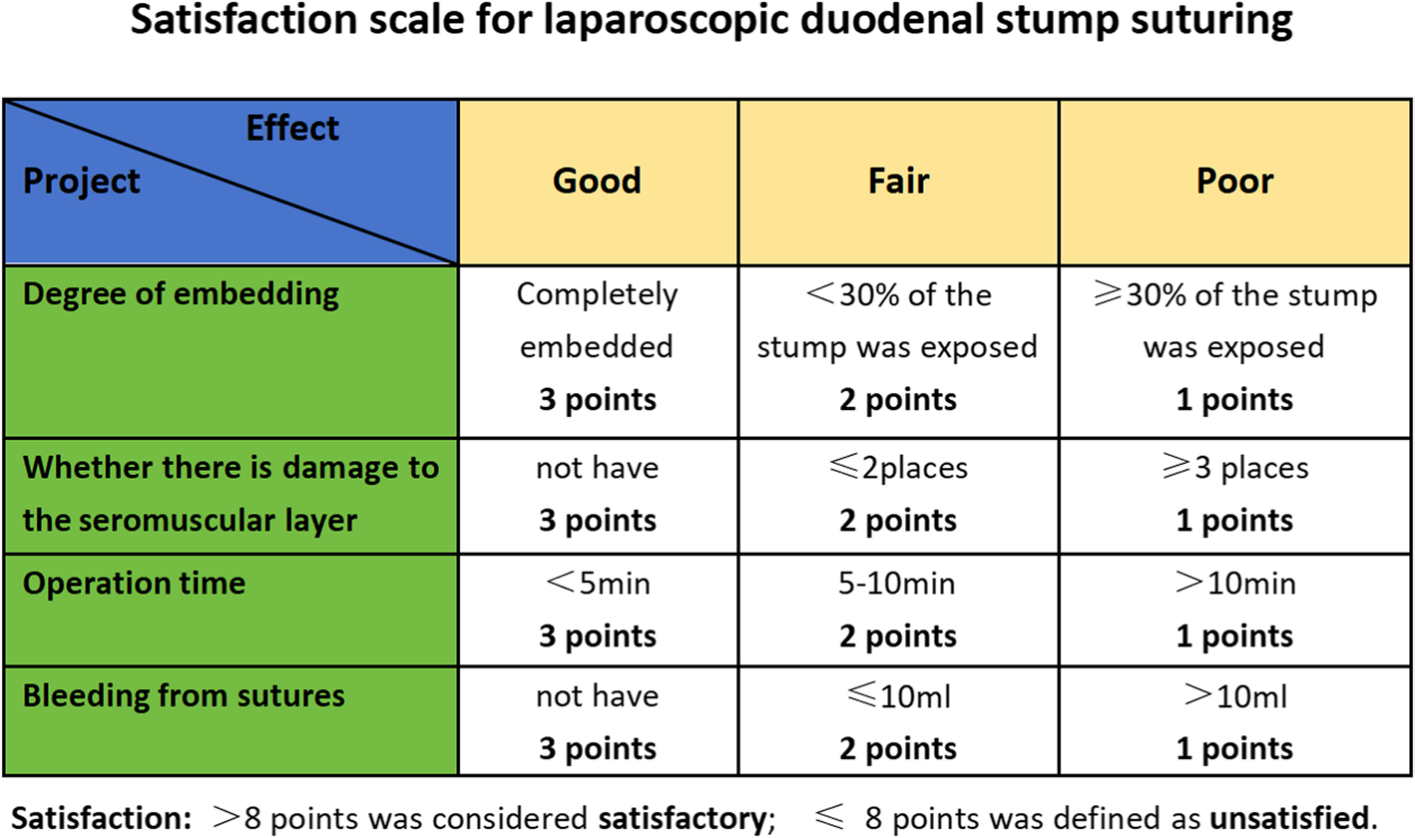
Satisfaction scale for laparoscopic duodenal stump suturing

Hospital and outpatient systems and telephone interviews gathered patient's demographics and clinicopathological characteristics. Demographics and clinicopathological characteristics included gender, Age, Body Mass Index (BMI), Hemoglobin, Albuminprior, Abdominal surgery history, Tumor diameter, Tumor location, The extent of resection, Types of reconstruction, American Society of Anesthesiologists (ASA) risk score, and tumor stage(NCCN Clinical Practice Guidelines in Oncology) [17]; Contrast data between not reinforced group and the reinforced group included Operative data, Pathologic data, Postoperative recovery data, and Related complications. Contrast data between QM group and CM group included operative time for Total gastrectomy, operative time for Distal gastrectomy, time for Purse-String Suture, a Success rate of the first purse-string suture, the Satisfaction rate (Fig. 4) of purse string suture. Analyzing and comparing the incidence of Duodenal Stump Leakage, reoperation rates for duodenal stump leakage, and reoperation-related mortality for duodenal stump leakage between the two subgroups. Duodenal Stump Leakage (DSL) refers to the leakage occurring at the duodenal stump after surgery, leading to the leakage of digestive fluid or other gastrointestinal contents into the abdominal cavity or surrounding tissues. The diagnosis of duodenal stump leakage is based on one or more of the following: 1) Clinical evidence of biliary drainage in the drain tube or abdominal wound, without other evidence of anastomotic leakage. 2) Computed tomography (CT) scan showing bile collection around the duodenal stump, followed by bile-stained aspiration or drainage.

3) Duodenography showing extravasation of contrast agent or duodenal fistula on contrast-enhanced imaging. 4) Intraoperative identification of duodenal leakage during surgical exploration [18].

### Statistical analysis

Normally distributed continuous variables were expressed as mean ± standard deviation and analyzed by an independent t-test. Non-normally distributed continuous variables were reported as the median and interquartile range and examined with non-parametric tests. Frequency variables were expressed as n (%) and analyzed using the chi-square or Fisher's exact test. Statistical analyses were performed using SPSS software (version 27.0). A two-tailed p-value less than 0.05 was considered statistically significant.

## Results

### Study protocol is illustrated in Fig. 5

**Fig. 5:**
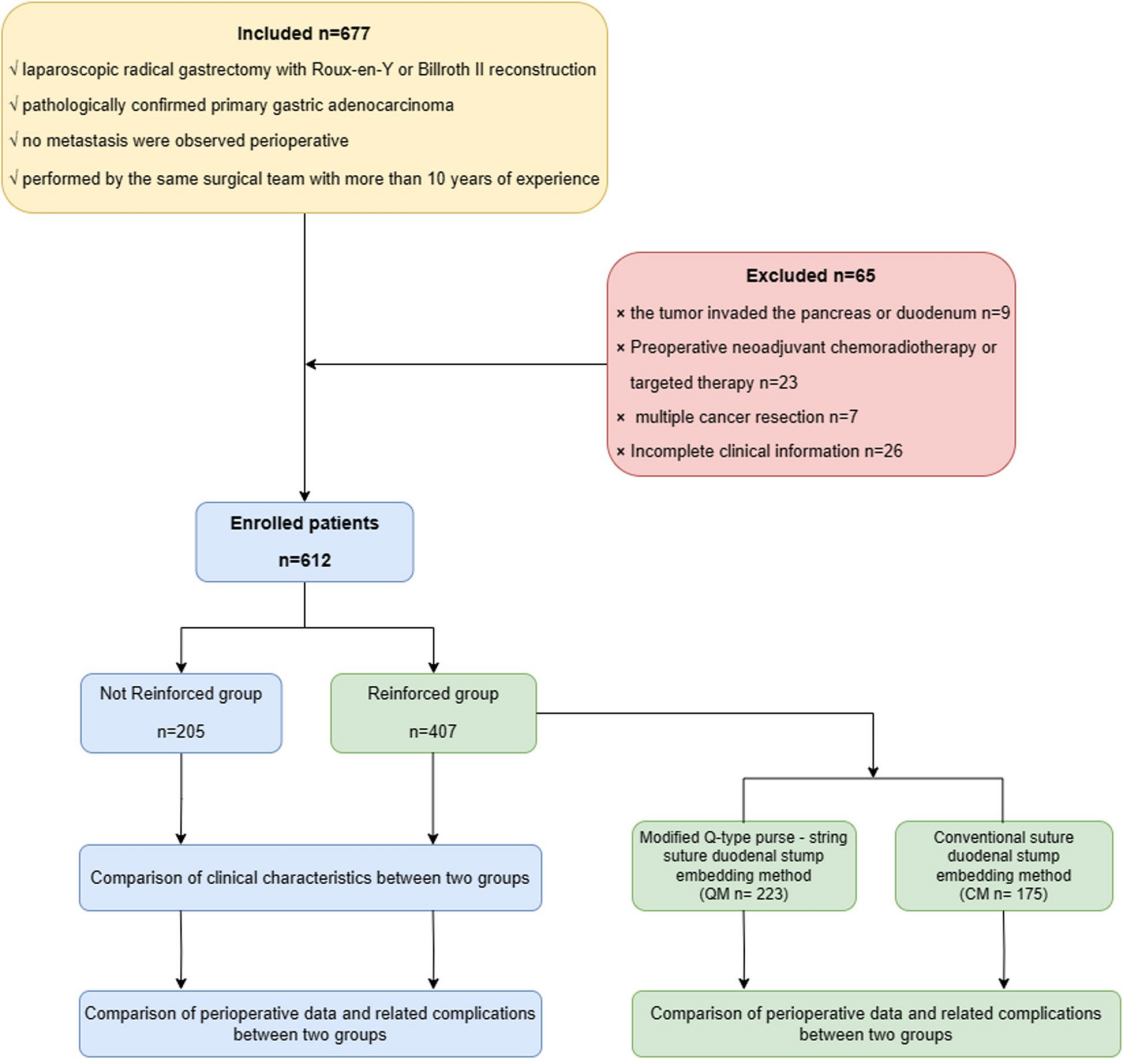
Study flow chart

#### Clinical and pathological characteristics (Table 1)

**Table 1:** Patient demographics and clinicopathological characteristics

Factor	Not Reinforced	Reinforced	Volume	p
Number of cases, n	205	407		
Gender			χ^2^ = 0.004	0.947
Male, n(%)	128(62.4%)	253(62.2%)		
Female, n(%)	77(37.6%)	154(37.8%)		
Age, years	67.90 ± 10.2	69.22 ± 9.268	t = -1.615	0.107
BMI, kg/m2, median (range)	24.4(22.4 ~ 26.1)	24.4(22.7 ~ 26.6)	z = -0.946	0.344
Hb, g/L, median (range)	124.1(117.4 ~ 131.7)	124.2(116.2 ~ 131.7)	z = -0.782	0.434
Alb, g/L, median (range)	38.8(36.6 ~ 41.4)	38.6(36.3 ~ 41.3)	z = -1.591	0.112
chronic disease				
Diabetes, n(%)	28(13.7%)	51(12.5%)	χ^2^ = 0.154	0.695
Hypertension, n(%)	46(22.4%)	85(20.9%)	χ^2^ = 0.196	0.658
pulmonary disease, n(%)	12(5.9%)	22(5.4%)	χ^2^ = 0.052	0.819
History of abdominal surgery, n(%)	24(11.7%)	61(15.0%)	χ^2^ = 1.227	0.268
Tumor diameter, cm, median (range)	2.8(1.6 ~ 4.2)	2.8(1.5 ~ 4.3)	z = -0.665	0.506
Tumor Location, n			χ^2^ = 3.341	0.188
Upper	89(43.4%)	206(50.6%)		
Middle	41(20.0%)	79(19.4%)		
Distal	75(36.6%)	122(30.0%)		
Extent of resection			χ^2^ = 2.729	0.099
Total gastrectomy	130(63.4%)	285(70.0%)		
Distal gastrectomy	75(36.6%)	122(30.0%)		
Types of reconstruction			χ^2^ = 2.729	0.099
Roux-en-Y	130(63.4%)	285(70.0%)		
Billroth-II	75(36.6%)	122(30.0%)		
ASA risk score, n(%)			χ^2^ = 3.615	0.164
I	63(30.7%)	105(25.8%)		
II	111(54.1%)	217(53.3%)		
III	31()15.1%	85(20.9%)		
Tumor stage, n(%)			χ^2^ = 3.876	0.275
I	18(8.8%)	45(11.1%)		
II	55(26.8%)	91(22.4%)		
III	108(52.7%)	236(58.0%)		
IV	24(11.7%)	35(8.6%)		

*BMI* Body Mass Index

*Hb* Hemoglobin

*Alb* Albumin

No statistically significant differences were observed in patient demographics and clinicopathological characteristics between the Not Reinforced Group and the Reinforced Group, indicating comparable baseline characteristics between the two groups.

### Study results

#### Perioperative-related indicators between the reinforced group and not reinforced group (Table 2)

**Table 2:** Perioperative-related indicators between the Reinforced group and Not Reinforced group

perioperative-related indicators	Not Reinforced (*n* = 205)	Reinforced (*n* = 407)	Volume	p
Operative data				
operative time for Total gastrectomy, min, median(range)	125(107 ~ 154)	130(105 ~ 161)	z = -0.777	0.437
operative time for Distal gastrectomy, min, median(range)	181(162 ~ 205)	188(167 ~ 206)	z = -1.645	0.100
time for reinforcement of stapled duodenal stump, min, ± SD	-	4.948 ± 1.9813	-	-
Blood loss, ml, median(range)	123(92 ~ 159)	125(90 ~ 160)	z = -0.031	0.975
Pathologic data				
Distance from distal resection margin for Distal gastrectomy (cm) (± SD)	4.8 ± 2.14	5.1 ± 2.30	t = -0.669	0.504
Retrieved lymph nodes, n, median(range)	33(19 ~ 44)	32(21 ~ 45)	z = -0.688	0.492
Positive margins, n (%)	2(1.0%)	3(0.7%)	χ^2^ = 0.096	0.757
Postoperative recovery				
First flatus, d, median(range)	2(1 ~ 3)	2(1 ~ 3)	z = -0.107	0.915
First exhaust, h, median(range)	49(41 ~ 56)	50(40 ~ 59)	z = -0.305	0.76
Postoperative hospital stay, days, (± SD)	10.90 ± 3.664	10.42 ± 2.524	t = 1.906	0.057
Related complications				
Intra-abdominal infection	4(2.0%)	7(1.7%)	χ^2^ = 0.041	0.839
Wound infection	9(4.4%)	8(2.0%)	χ^2^ = 2.968	0.085
Bleeding	2(1.0%)	3(0.7%)	-	0.757
obstruction	5(2.4%)	10(2.5%)	χ^2^ = 0.012	0.913
Fluid collection and abscess	4(2.0%)	10(2.5%)	χ^2^ = 0.156	0.693
Pancreatic leakage ^a^	5(2.4%)	16(3.9%)	χ^2^ = 0.916	0.339
Anastomotic leakage	4(2.0%)	7(1.7%)	χ^2^ = 0.041	0.839
Duodenal stump leakage	5(2.4%)	3(0.7%)	-	0.126
Reoperation for duodenal stump leakage	2(1.0%)	0	-	0.046*
Reoperation for duodenal stump leakage-related death	1(0.5%)	0	-	0.158
Clavien–Dindo morbidity ^b^			χ^2^ = 1.802	0.406
I–II, n (%)	38(18.5%)	60(14.7%)		
III–IV, n (%)	3(1.5%)	4(1.0%)		

^a^ Pancreatic leakage: Follow the International Study Group on Pancreatic Fistula (ISGPF) leak criteria [19], ≥ 3 days, amylase 3 × normal

^b^ Clavien–Dindo morbidity: Follow the Clavien-Dindo classification of surgical complications [20]

^*^ Denotes statistical signifcance with *p* < 0.05

In terms of total operative time, the Not Reinforced Group demonstrated a slightly shorter median operative time compared to the Reinforced Group [Distal gastrectomy: 125 (107 ~ 154) vs 130 (105 ~ 161) min (*p* = 0.437), Total gastrectomy: 181 (162 ~ 205) vs 188 (167 ~ 206) min (*p* = 0.1)]. These differences were not statistically significant. The incidence of duodenal stump leakage in the Not Reinforced Group [2.4% (5/205)] was higher than that in the Reinforced Group [0.7% (3/407)], but the difference was not statistically significant (*p* = 0.126). In the Not Reinforced Group, two patients required a second surgery due to duodenal stump leakage, resulting in a higher reoperation rate compared to the Reinforced Group [2 (1.0%) vs. 0, *p* = 0.046], and this difference was statistically significant. One patient in the Not Reinforced Group succumbed to duodenal stump leakage, leading to a mortality rate higher than that in the Reinforced Group [1 (0.5%) vs. 0, *p* = 0.158], but the difference was not statistically significant. No significant differences were observed between the two groups in terms of operative data, pathologic data, postoperative recovery, and related complications.

#### Subgroup analysis of the reinforced group (Tables 3 and 4)

**Table 3:** Patient demographics and clinicopathological characteristics between the QM group and CM group

Factor	QM (*n* = 232)	CM (*n* = 175)	Volume	p
Gender			χ^2^ = 1.146	0.283
Male, n(%)	149(64.2%)	104(59.4%)		
Female, n(%)	83(35.8%)	71(40.6%)		
Age, years	67.05 ± 9.49	65.96 ± 10.03	t = 1.359	0.233
BMI, kg/m2, median (range)	24.4(22.3 ~ 26.7)	24.1(22.3 ~ 26.5)	z = -1.141	0.253
Hb, g/L, median (range)	123.3(116.3 ~ 132.4)	124.2(115.2 ~ 132.6)	z = -0.427	0.667
Alb, g/L, median (range)	38.7(36.3 ~ 41.5)	38.5(36.1 ~ 41.2)	z = 0.243	0.804
chronic disease				
Diabetes, n(%)	27(11.6%)	25(14.3%)	χ^2^ = 0.981	0.319
Hypertension, n(%)	41(17.7%)	38(21.7%)	χ^2^ = 1.173	0.275
pulmonary disease, n(%)	14(6.0%)	8(4.6%)	χ^2^ = 0.389	0.536
History of abdominal surgery, n(%)	34(14.7%)	21(12.0%)	χ^2^ = 0.853	0.354
Tumor diameter, cm, median (range)	2.7(1.3 ~ 4.2)	2.8(1.2 ~ 4.4)	z = -0.726	0.463
Tumor Location, n			χ^2^ = 0.945	0.615
Upper/Middle/Distal	77/47/108	57/42/76		
Extent of resection			χ^2^ = 0.375	0.527
Total gastrectomy/Distal gastrectomy	123/109	98/76		
Types of reconstruction			χ^2^ = 0.375	0.527
Roux-en-Y/ Billroth-II	123/109	98/76		
ASA risk score, n(%)			χ^2^ = 4.027	0.131
I/II/III	62/118/52	52/97/26		
Tumor stage, n(%)			χ^2^ = 3.958	0.234
I/II/III/IV	30/74/128/0	17/71/87/0		

*BMI* Body Mass Index, *Hb* Hemoglobin, *Alb* Albumin

**Table 4:** Perioperative-related indicators between the QM group and CM group

Perioperative-related indicators	QM (*n* = 232)	CM (*n* = 175)	Volume	p
Operative time for Total gastrectomy, min, median(range), min	128(103 ~ 158)	131(104 ~ 165)	z = -0.655	0.547
Operative time for Distal gastrectomy, min, median(range)	186(160 ~ 203)	190(168 ~ 210)	z = -1.212	0.203
Duodenal stump reinforcement time, min	4.11 ± 1.840	6.05 ± 1.577	t = -11.225	0.001*
Initial success rate of burying the duodenal stump, n	216(93.1%)	136(77.7%)	χ^2^ = 20.214	0.001*
Satisfaction rate of purse string suture, n	224(96.6%)	157(89.7%)	χ^2^ = 7.798	0.005*
Duodenal stump leakage, n	0	2(1.1%)	χ^2^ = 2.665	0.103
Reoperation for duodenal stump leakage, n	0	0	-	-
Reoperation for duodenal stump leakage-related death, n	0	0	-	-

Initial success rate of burying the duodenal stump: This metric is determined by retrospectively reviewing the surgical video footage of each enrolled patient, observing the process of burying the duodenal stump. It involves counting the number of cases where complete burying was achieved during the first suturing

Satisfaction rate of purse string suture: This assessment is performed using the Satisfaction scale for laparoscopic duodenal stump suturing, developed through comprehensive evaluation at our center (scale displayed in Fig. 4)

^*^ Denotes statistical signifcance with *p* < 0.05

There were no statistically significant differences in demographic and clinical pathological data between the two groups, indicating comparability (Table 3). There were no statistically significant differences between the two groups in terms of total operative time. However, the QM subgroup demonstrated superiority over the CM subgroup in duodenal stump reinforcement time (4.11 ± 1.840 vs. 6.05 ± 1.577, *p* = 0.001), the initial success rate of burying the duodenal stump (93.1% vs. 77.7%, *p* = 0.001), and duodenal stump burial satisfaction rate [224 (96.6%) vs. 157 (89.7%), *p* = 0.005], with statistically significant differences. No occurrences of duodenal stump leakage were reported in the QM subgroup, while two cases occurred in the CM subgroup [2 (1.1%)], but the difference between the two subgroups was not statistically significant. Both subgroups did not experience secondary surgeries or deaths related to duodenal stump leakage.

## Discussion

In patients undergoing gastric cancer surgery, such as fistula [21], infection, obstruction, thrombotic diseases [22], etc. Duodenal Stump Leakage (DSL) is a serious complication [9], albeit rare, with a conservative treatment failure rate of up to 30% and a mortality rate of 28%. Currently, there is a lack of standardized treatment methods applicable to all cases. Studies indicate that laparoscopic gastric cancer surgery increases the risk of DSL, leading to exploration of various methods for reinforcing the duodenal stump. However, there is conflicting evidence regarding the necessity of routine duodenal stump reinforcement. In this context, our center has modified traditional reinforcement methods and proposed an modified Q-type purse-string suture duodenal stump embedding technique. It has been found that laparoscopic reinforcement of the duodenal stump can reduce the severity of fistulas and the probability of reoperation. The modified Q-type purse-string suture duodenal stump embedding method is simple and effective, reducing surgical time, improving satisfaction, and lowering the incidence of DSL to some extent.

Standardized surgical techniques offer the advantage of procedural consistency, forming the foundation for safe and efficient surgical execution. Previous studies have identified several independent risk factors for DSL, including preoperative pyloric obstruction leading to duodenal inflammation [23], elevated preoperative C-reactive protein (CRP) [24], obesity [24], lack of reinforcement at the duodenal stump [24], afferent loop obstruction [23, 25], and D2 lymph node dissection combined with subtotal gastrectomy [26]. Reinforcement of the duodenal stump serves as a protective factor against duodenal stump leakage. Numerous studies have explored various reinforcement techniques, such as the Bioabsorbable Polyglycolic Acid (BPA)sheet + fibrin sealant proposed by Paik et al. [23], BPA sheet by Misawa et al. [27], Lembert Suture by Inoue et al. [14], Barbed suture by Kim MC et al. [15], Single Purse-String Suture by He et al. [28], Handover method by Du et al. [29], and Buried suture by Ri et al. [30]. These methods have demonstrated preventive effects against DSL. However, there is currently no standardized reinforcement method, and clinical studies comparing these approaches are lacking.

Simultaneously, it is important to recognize that DSL is a rare complication. Our study statistics indicate an incidence rate of DSL at 1.3%, which aligns with previous research findings (1.6%—5%) [9, 10]. There is currently no consensus regarding the necessity of reinforcing the duodenal stump to prevent DSL. Some studies suggest that duodenal stump reinforcement can reduce the occurrence of DSL [12], while others show that the reinforcement's preventive effect on DSL is not significant [15, 31]. Our study results indicate that, although the incidence rate of DSL decreased in the reinforced group compared to the unreinforced group (0.7% vs. 2.4%), there was no statistically significant difference, possibly due to the lower DSL incidence, which did not demonstrate statistical significance. The findings of this study suggest that reinforcing the duodenal stump can reduce the rate of Reoperation following DSL occurrence.

Duodenal stump reinforcement involves intricate maneuvers in a confined space, and existing research indicates varying impacts on overall operative duration [12, 28, 29]. This suggests differences among centers in the operational details of duodenal stump reinforcement, with the significantly extended operative duration highlighting the complexity of this procedure. In this study, we standardized the operational procedure for duodenal reinforcement, with the Q-type suture group having a final operation time of (4.11 ± 1.840 min). Although the total operative time was slightly prolonged, there was no statistically significant difference. Furthermore, in terms of postoperative recovery indicators and related complications, there was no statistically significant difference between the reinforced group and the unreinforced group, confirming the safety of duodenal stump reinforcement. Our subgroup analysis indicates that the Q-type suture is a straightforward, safe, and feasible method for duodenal stump reinforcement.

Research findings indicate that reinforcing the duodenal stump can reduce the risk of severe complications following delayed gastric emptying [30]. Consistent with these results, our study has found several similar ones, based on which we routinely perform laparoscopic duodenal stump embedding during laparoscopic radical gastrectomy for gastric cancer.

**Fig. 6:**
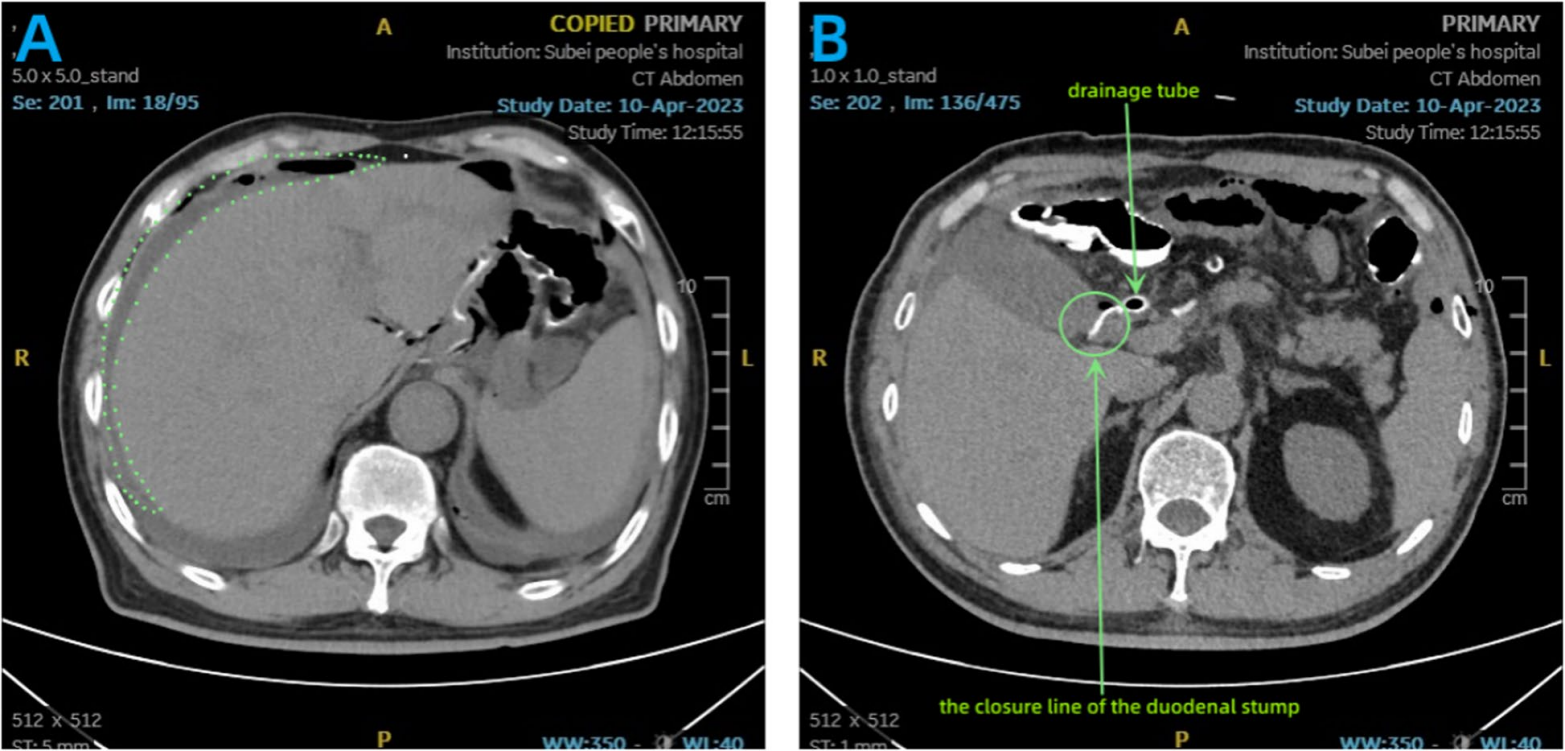
**A **Abdominal CT Scan of a Patient with Duodenal Stump Fistula: The dashed lines indicate the location where leaked fluids and gas accumulate around the liver. **B** Closure Line of Duodenal Stump in this DSL Patient: The closure line of the duodenal stump is depicted in the figure. It appears as a complete and intact line without any apparent defects

Prior studies have indicated that in patients who develop DSL, the closure line of the duodenal stump remains intact [30]. This finding aligns with our research results (Fig. 6A, B), suggesting that the occurrence of DSL may be associated with excessive detachment of the residual stump vessels, leading to defects in the serosal layer [32] and an increase in tension within the residual peristaltic cavity. Therefore, we consider the burial of the stump as an effective measure for preventing DSL. Compared to methods such as the use of barbed suture continuous closure or absorbable interrupted suture closure, pouch suture closure undoubtedly represents a stump reinforcement technique with the fewest stitches and the most time-saving approach. Another noteworthy aspect is the success rate and satisfaction level of the procedure, which has not been previously addressed in the literature.

**Fig. 7:**
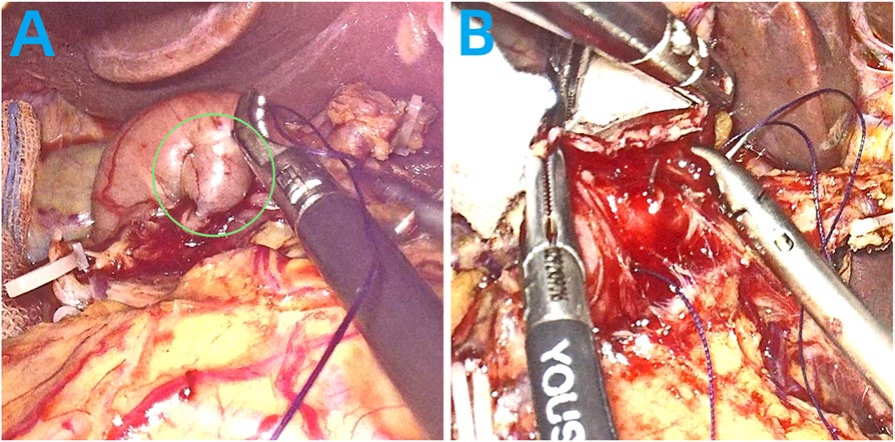
**A** Incomplete Burial of the Duodenal Stump with Exposed Angle. **B** Intraoperative Hemorrhage Caused by Inadvertent Suturing

Considering the potential for procedural failure and aiming to further objectively assess the advantages and disadvantages of the Q-type suture compared to the conventional suture burial method, this study introduced innovative metrics: suture satisfaction rate (Fig.4) and the first-attempt suture success rate. A retrospective analysis was conducted by reviewing surgical videos of all enrolled patients, documenting procedural time, procedural smoothness, and final burial satisfaction. In this study, the conventional method of duodenal stump burial had a duration of (6.05 ± 1.57 min), which was longer than the Q-type suture. This discrepancy can be attributed to the lower first-attempt success rate of the conventional method, where the two intersecting angles of the stump are prone to dislodgement (Fig.7A). The increased risk of bleeding due to inadvertent needle injury to vessels and the need for repeated procedures and hemostasis contribute to the extended surgical time(Fig. 7B). To enhance duodenal stump reinforcement at our center, modifications were implemented, specifying a total of 5 stitches. Starting points were chosen above and below the duodenal vascular bundle to avoid vessel injury. Collaborative efforts involving two individuals increased the first-attempt success rate. Following these improvements, the Q-type suture for duodenal stump burial had a reduced time of (4.11 ± 1.84 min), significantly shortening the procedural duration with statistically significant differences.

**Fig. 8:**
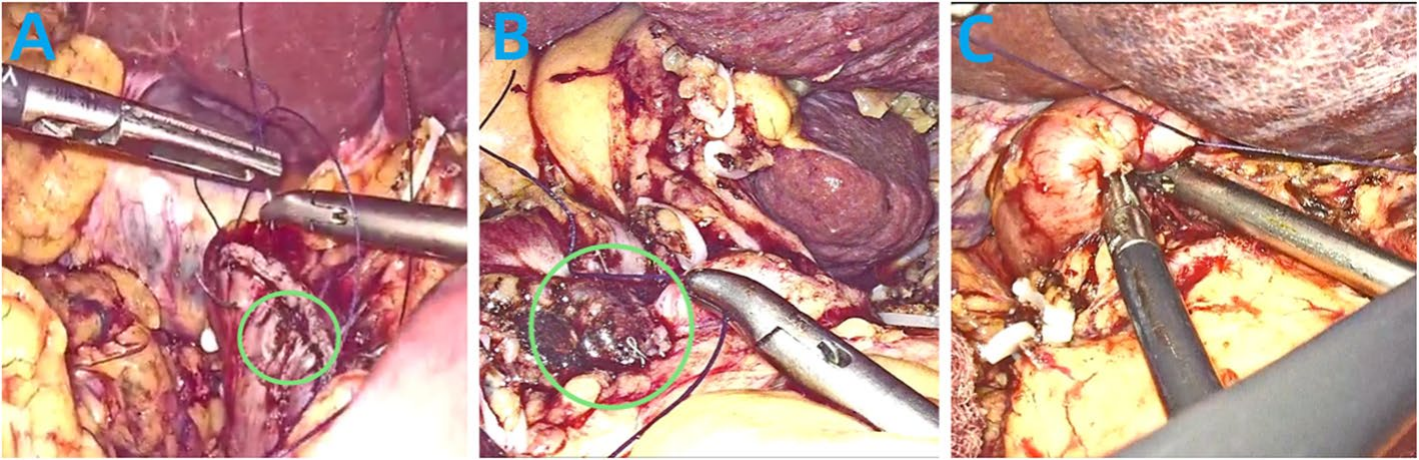
**A** Following unsuccessful burial, a remedial procedure involves full-layer continuous suturing reinforcement of the duodenal stump. Partial damage to the muscular layer of the duodenal stump is observed at certain locations. **B** Incomplete Burial of the Duodenal Stump with Exposed Angle. **C** Controlling the duodenal stump to stay within the pouch, the assistant tightens the suture, enhancing the success rate of burial

In this study, two cases of DSL occurred in the conventional duodenal reinforcement group. Postoperative video analysis revealed that in one case, duodenal stump burial failed, and as a salvage measure, further continuous suturing was performed to reinforce the duodenal stump (Fig. 8A). This patient developed duodenal stump leakage six days postoperatively but showed improvement after conservative treatment with measures including fasting, administration of somatostatin, intravenous nutritional support, and effective drainage. In another case, the duodenal stump burial result was suboptimal, with incomplete burial and residual exposure of the stump (Fig. 8B). This patient experienced DSL seven days postoperatively, with subsequent improvement after conservative treatment.

In contrast, the modified Q-type suture method did not result in anastomotic leakage, and this difference was statistically significant. These findings indicate that while reinforcing the duodenal stump can reduce the risk of severe complications associated with duodenal stump leakage, unsuccessful or incomplete burial does not effectively prevent the occurrence of duodenal stump leakage. This also emphasizes the advantage of the Q-type purse-string suture burial method, wherein the surgeon's hands are involved in burying the duodenal stump into the purse-string, with assistance from the assistant in tightening the suture line (Fig. 8C), significantly improving the success rate and satisfaction of duodenal stump burial.

In summary, DSL is a severe but rare complication. There is no statistically significant relationship between duodenal stump reinforcement and the incidence of DSL. However, laparoscopic reinforcement of the duodenal stump can reduce the severity of fistulas and the probability of Reoperation. The laparoscopic modified Q-type purse-string suture burial method for reinforcing the duodenal stump is simple and effective. It can, to a certain extent, reduce the operating time, enhance purse-string burial satisfaction, show a trend of reducing the incidence of DSL, and improve patient prognosis to some extent.

### Supplementary Information

**Supplementary Material 1.**

## Data Availability

The data that support the findings of this study are available from the corresponding author upon reasonable request.
